# A Molecular Study on Hepatitis E Virus (HEV) in Pigs in Bulgaria

**DOI:** 10.3390/vetsci8110267

**Published:** 2021-11-06

**Authors:** Andrea Palombieri, Ilia Tsachev, Vittorio Sarchese, Paola Fruci, Federica Di Profio, Roman Pepovich, Magdalena Baymakova, Fulvio Marsilio, Vito Martella, Barbara Di Martino

**Affiliations:** 1 Faculty of Veterinary Medicine, Università degli Studi di Teramo, 64100 Teramo, Italy; apalombieri@unite.it (A.P.); vsarchese@unite.it (V.S.); pfruci@unite.it (P.F.); fdiprofio@unite.it (F.D.P.); fmarsilio@unite.it (F.M.); 2 Department of Microbiology, Infectious and Parasitic Diseases, Faculty of Veterinary Medicine, Trakia University, 6000 Stara Zagora, Bulgaria; ilia_tsachev@abv.bg; 3 Department of Infectious Pathology, Hygiene, Technology and Control of Foods from Animal Origin, Faculty of Veterinary Medicine, University of Forestry, 1797 Sofia, Bulgaria; rpepovich@abv.bg; 4 Department of Infectious Diseases, Military Medical Academy, 1606 Sofia, Bulgaria; dr.baymakova@gmail.com; 5 Department of Veterinary Medicine, Università Aldo Moro di Bari, 70010 Valenzano, Italy; vito.martella@uniba.it

**Keywords:** hepatitis E virus (HEV), RNA, genotype 3, pigs, Bulgaria

## Abstract

Information on hepatitis E virus (HEV) strains circulating in animal reservoirs in Bulgaria is currently lacking. Herein, by screening HEV seropositive sera obtained from Bulgarian swine and wild boars, viral RNA was detected at high prevalence rate (28.2%) in industrial pigs. Sequence analysis of the partial polymerase (RdRp) region revealed the highest genetic correlation with HEVs of genotype (Gt) 3 identified in French and Dutch patients. For three such strains, a 700-bp fragment of the open reading frame 2 gene was generated. On phylogenetic analysis, the Bulgarian strains clustered tightly (93.8–98.3% nt) with human and animal HEVs classified within the Gt3 subtype c.

## 1. Introduction

Hepatitis E virus (HEV) is the main cause of acute viral hepatitis worldwide. Although HEV causes mostly self-limiting hepatitis, infection in immunosuppressed patients such as organ transplant recipients can progress into chronicity [1]. The possible association between HEV infection and extrahepatic manifestations including neurological and renal injuries has been also hypothesized [2]. HEV, classified in the family *Hepeviridae*, is a single-stranded, positive-sense RNA virus of ~7.2 kb in length that in the infected host exists in two distinct forms, as non-enveloped (neHEV) particle of approximately 27–34 nm in diameter when secreted in the feces [3], or as quasi-enveloped (eHEV) virion in circulating blood and in the supernatant of infected cell cultures [4]. HEVs of mammalian origin are classified along with avian strains into the genus *Orthohepevirus*, comprising four species, *Orthohepevirus A-D* [5]. The species *Orthohepevirus A* includes at least eight distinct genotypes (Gts), with four major Gts (1–4) implicated in human disease. Gt1 and Gt2 infections are restricted to humans and cause large epidemics in developing countries due to poor sanitation and lack of clean drinking water, while Gt3 and Gt4 are zoonotic and cause sporadic and cluster cases of hepatitis E in both industrialized and developing countries [6]. In the last few years, a steady increase in cases of human HEV infection has been observed in Europe, mostly of autochthonous origin [7]. In Bulgaria, the burden of disease and transmission pathways for hepatitis E cases have not been sufficiently defined yet. However, accumulated data suggest that HEV infection could represent a public health problem in local populations. A recent serosurvey conducted on Bulgarian blood donors reported an overall HEV IgG prevalence of 25.9% (144/555), with regional differences ranging from 21.3% to 28.8% [8]. Furthermore, a critical analysis from 23 independent Bulgarian studies, which combined HEV IgG, IgM and RNA markers from 2257 hospitalized patients with clinical presentation of acute liver injuries, estimated that in the time frame 1995–2018 the overall HEV infection prevalence was 13.1% [9]. HEV RNA was found in 90 (87.3%) out of 103 sera collected between January 2013 and May 2015 from patients with diagnosed hepatitis E. Upon sequence analysis of a subset (64/90) of positive sera, Gt3 HEV RNA was identified in 98.4% (63/64) of the assessed samples [10]. In similar fashion, serological surveys on animal reservoirs have revealed prevalence rates ranging from 40.0% to 60.3% in domestic swine [11,12,13], to 40.8% in wild boars [14] and to 82.5% in East Balkan swine (autochthonous pig breed) [15]. Although these data unequivocally demonstrated a high viral circulation in pigs and wild boar local populations, information on HEV molecular epidemiology has not been collected, thus hindering the depiction of a complete portrait of the impact that animal reservoirs may have in the transmission of infection to humans. The aim of the present study was to molecularly investigate the circulation of HEV in pigs and wild boars from Bulgaria and to characterize the strains detected.

## 2. Materials and Methods

### 2.1. Sampling

Molecular screening was performed on a total of 39 serum samples obtained from HEV seropositive pigs and wild boars collected between January 2018 and February 2020 in Bulgaria [12,13,14,15]. In detail, ten sera were collected from fattening pigs bred in an industrial farrow-to-finish farm in Vidin district (Northern Bulgaria), an additional ten were from fattening pigs farmed in an industrial Southern Bulgaria herd located in the Yambol district, and a further group of nine sera was collected from fattening East Balkan swine in Burgas district (Eastern Bulgaria). Furthermore, 10 sera were collected from wild boars during the official hunting season in the western district (Blagoevgrad) of Bulgaria. 

### 2.2. Molecular Screening

Total RNA was extracted from 200 μL of each serum by using the ExgeneTM Viral DNA/RNA mini (TEMA Ricerca, Bologna, Italy), following the manufacturer’s instructions. The presence of *Orthohepevirus A* RNA was assessed by real-time reverse transcription PCR (qRT-PCR), targeting a conserved 68 nucleotide region of ORF3 gene, as previously described [16]. An HEV plasmid was constructed by cloning the 68 bp ORF3 fragment of a wild boar strain (GenBank accession no. KU508285) [17] into the Topo TA cloning vector (Invitrogen, Ltd., Milan, Italy). Tenfold serial dilutions of the plasmid ranging from 10^9^ to 10^0^ copies per reaction were used as the standard curve in each PCR run. Additionally, molecular screening was carried out by heminested RT-PCR using *Hepeviridae* broadly reactive primers targeting a region of 338-bp of the viral RNA-dependent RNA polymerase (RdRp) complex [18]. All the positive samples were further tested by RT-PCR and nested PCR to amplify, respectively, fragments of 755 bp and 348 bp of the ORF2 gene [19]. Primer sets used in this study are listed in Table 1. 

### 2.3. Sequence Analysis

Amplicons were excised from the gel and purified using the QIAquick gel extraction kit (Qiagen GmbH, Hilden, Germany). Each fragment was then subjected to bidirectional Sanger sequencing using BigDye Terminator Cycle chemistry and 3730 DNA Analyzer (Applied Biosystems, Foster, CA, USA). Basic Local Alignment Search Tool (BLAST; http://www.ncbi.nlm.nih.gov, accessed on 15 October 2021) and FASTA (http://www.ebi.ac.uk/fasta33, accessed on 15 October 2021) with default values were used to find homologous hits. The alignment of the sequences was conducted using the MAFFT multiple alignment program version 7.388 plugin of the Geneious Prime software V. 21.1.1 (Biomatters Ltd., Auckland, New Zealand). Phylogenetic analysis was conducted using Maximum Likelihood, Tamura-Nei model, supplying statistical support with bootstrapping of 1000 replicates, in MEGA X software [20].

## 3. Results

On molecular screening by qRT-PCR of 39 serum samples, HEV RNA was detected in a total of 11 sera (28.2%; 11/39) (Table 2) with viral loads ranging from 1.5 × 10 ^3^ to 7.0 × 10 ^4^ RNA copies/mL of template (mean 2.6 × 10 ^4^ RNA copies/mL). Out of 11 positive specimens, 10 (90.9%; 10/11) were identified in pigs from Northern Bulgaria and only one (10.0%; 1/10) was found in a pig from the industrial Southern Bulgaria herd. None of the sera from East Balkan swine and from wild boars tested positive for HEV RNA. When re-testing the sera with pan-hepeviruses primer pairs [18], nine samples all from the collection from the Northern Bulgaria farm (90.0%; 9/10) yielded a band of expected size (Table 2).

**Figure 1:**
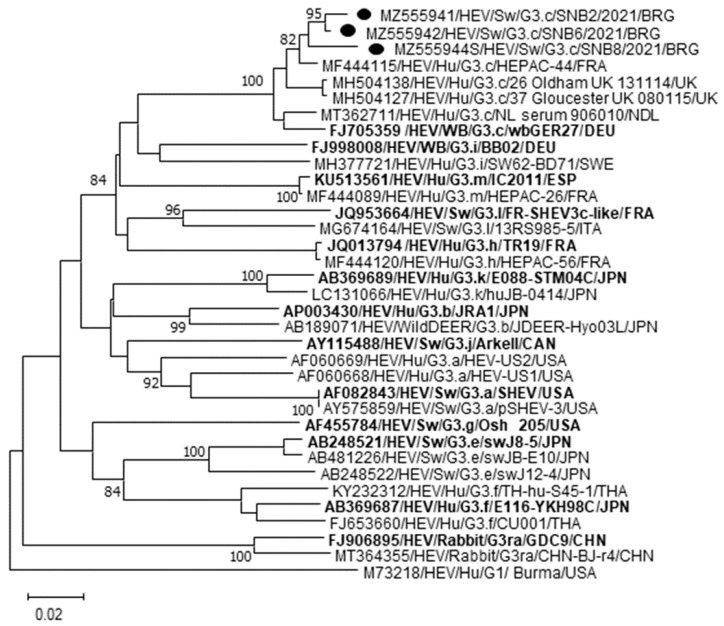
Phylogenetic tree constructed on the partial ORF2 gene of three Bulgarian HEV swine strains (GenBank accession no. MZ555941, MZ555942 and MZ555944). Tree was generated using Maximum Likelihood method based on the Tamura-Nei model and supplying statistical support with bootstrapping of 1000 replicates. The scale bar indicates nucleotide substitutions per site. The reference strains representative of each HEV Gt3 subtype are in boldface. The marker denotes the HEV sequences detected in this study. Evolutionary analyses were conducted in MEGA X.

Partial RdRp sequences were determined and deposited in GenBank under the accession numbers MZ519907-MZ519915. Upon BLAST and FASTA analyses, the amplicons shared 99.4–100% nucleotide (nt) identity with each other and displayed the closest relatedness (96.4–98.8%) to human HEV Gt3 strains previously detected in serum samples from French patients (MW355218, MW355367, MW355268, MW355295) [21] and in solid organ transplant recipients in the Netherlands (JQ015416 and JQ015424) [22]. The ORF2 sequence of ~ 700 nt was obtained for the strains SNB2, SNB6, and SNB8 (MZ555941–42 and MZ555944), whilst one additional specimen, HEV SNB7 (MZ555943), yielded a positive result after second-round amplification in nested PCR (~348 nt) (Table 2). In the ORF2-based tree, performed on the region of 700 nt, the Bulgarian strains segregated within the clade 1 (abchkijlm) [23,24,25] displayed the closest identity (93.8–98.3%) with animal and human HEVs assigned to subtype c. Identities related to the other Gt3 subtypes were from 81.6% (Gt3ra) to 88.9% (Gt3i) (Figure 1).

We next compared the ORF2 sequences detected in this study with the sequences of 63 Bulgarian human Gt3 HEV strains [10] in the overlapping ~240 nt portion. The overall nucleotide identity ranged from 80.1% to 93.9%, with the best match (92.0–93.9%) to the strains ISS2/Sof2013 and ISS76/Dob/2014 (MH203165 and MH203204) classified as subtype c (Table 3).

## 4. Discussion

In the last few years, improved surveillance activities in Bulgaria have shown that zoonotic HEV infection is emerging [8,9,10]. In this study, to provide insights into the molecular epidemiology of HEV in animal reservoirs, we screened, by a multitarget gene approach, a collection of sera from Bulgarian swine and wild boars previously found positive for HEV IgG antibodies. HEV RNA was detected in 28.2% (11/39) of the animals tested. Out of the four collections assessed in this study, the highest rate (100%; 10/10) was found in pig sera obtained from a closed-cycle industrial farm in North Bulgaria, in which specific HEV antibodies were detected with a prevalence of 63.3% (19/30). It could be hypothesized that at the sampling time point most of the pigs were either seropositive or viremic. Additionally, sequence analysis of the RdRp fragment (338 nt) generated for nine strains revealed a clonal origin (99.4–100% identity) that may be compatible with the circulation of the same variant within the herd investigated. Only one sample from the industrial Southern Bulgaria herd produced a positive result by quantitative RT-PCR but attempts made to obtain sequence data failed. Furthermore, all serum samples collected from East Balkan swine and wild boars tested negative either when tested with quantitative or conventional PCR. A major limit of our investigation was the small number of samples included in the screening as we tested only serum samples available in our laboratories. Accordingly, it is likely that these results rather than mirroring the actual geographical distribution of HEV in Bulgaria, are merely linked to the limited number of animals analyzed or at least to the absence of viremic status at the time of sampling. In our study, sequence analysis of a 700-nt portion of ORF2 allowed a subtyping classification of the swine Bulgarian strains SNB2, SNB6 and SNB8, revealing the highest genetic correlation to HEV Gt3 subtype c. This cluster comprises HEV strains of human and animal origin frequently detected in Europe [25]. Accordingly, the spread of this subtype in Bulgaria could be accounted for by geographical changes in the circulation of HEV strains, likely due to trading of HEV-infected animals from other European countries. Data on the diffusion and distribution of HEV Gt3 subtypes circulating in Bulgaria in human population are still limited. Phylogenetic and coalescence analyses based on a 355-nt partial ORF2 region of 63 HEV Gt3 strains identified in patients with hepatitis E [10,11,12,13,14,15,16,17,18,19,20,21,22,23,24,25,26] provided evidence that subtypes 3e (39/63, 62%), 3f (15/63, 24%) and 3c (8/63, 13%) are the most common. In our analysis, we retrieved all the Bulgarian HEV human sequences from the GenBank database [10] and compared them with the porcine sequences generated in this study. On pairwise alignment of a 240-nt ORF2 overlapping fragment, the highest genetic correlation (92.0–93.9%) was found with two human sequences (ISS2/Sof2013 and ISS76/Dob/2014) both classified within the subtype c, confirming that similar HEVs are circulating in humans and animals in the same geographical setting.

## 5. Conclusions

In summary, our study provided a molecular confirmation of the presence of HEV in pigs from Bulgaria, documenting the identification of a subtype (c) previously detected in Bulgarian patients. As pointed out in this study, large-scale molecular surveillance of animal HEV is necessary to improve the knowledge on the strains circulating and to track the origin of HEV infection in humans. 

## Figures and Tables

**Table 1:** List of primers used in this study. Nucleotide position refers to the sequence of the strain HEV/SwGt3/Meng (GenBank accession no. AF082843).

Oligonucleotide	Position	Sequence (5′ to 3′)	Sense	Reference
JHEV FW	5285–5302	GGTGGTTTCTGGGGTGAC	+	[16]
JHEV REV	5337–5354	AGGGGTTGGTTGGATGAA	-	[16]
JHEV P	5308–5325	FAM-TGATTCTCAGCCCTTCGC-BHQ	+	[16]
HEV-F4228	4279–4307	ACYTTYTGTGCYYTITTTGGTCCITGGTT	+	[18]
HEV-R4598	4627–4649	GCCATGTTCCAGAYGGTGTTCCA	-	[18]
HEV-R4565	4591–4616	CCGGGTTCRCCIGAGTGTTTCTTCCA	-	[18]
HEVestFW	5711–5732	AAYTATGCWCAGTACCGGGTTG	+	[19]
HEVestREV	6419–6441	CCCTTATCCTGCTGAGCATTCTC	-	[19]
HEVintFW	5986–6017	GTYATGYTYTGCATACATGGCT	+	[19]
HEVintREV	6324–6343	AGCCGACGAAATYAATTCTGTC	-	[19]

**Table 2:** Molecular prevalence of HEV in sera collected form pigs and wild boars in Bulgaria.

Animal Collections	No. of Animals Tested	Positive/Total (%)			
		qRT-PCR ORF3 [16]	Heminested RT-PCR RdRp [18]	RT-PCR ORF2 [19]	Nested PCR ORF2 [19]
Swine Northern Bulgaria	10	10/10 (100.0)	9/10 (90.0)	3/10 (30.0)	4/10 (40.0)
Swine Southern Bulgaria	10	1/10 (10.0)	0/10 (0.0)	0/10 (0.0)	0/10 (0.0)
Swine Eastern Bulgaria	9	0/9 (0.0)	0/9 (0.0)	0/9 (0.0)	0/9 (0.0)
Wild boars	10	0/10 (0.0)	0/10 (0.0)	0/10 (0.0)	0/10 (0.0)
Total	39	11/39 (28.2)	9/39 (23.1)	3/39 (7.7)	4/39 (10.2)

**Table 3:** Nucleotide (nt) sequence identity matrix (%) based on the partial ORF2 gene of a selection of human HEV strains identified in Bulgaria [10]. The swine strains detected in this study are in bold.

HEV Gt3 Subtype	Strain Name	MZ555941 SNB2	MZ555942 SNB6	MZ555944 SNB8	GenBank Accession No.
**3c**	ISS2/Sof2013	93.9%	93.8%	92.3%	MH203165
	ISS76/Dob2014	93.5%	93.5%	92.0%	MH203204
	ISS75/Plov2014	89.4%	89.6%	88.5%	MH203203
	ISS92/Sof2014	88.2%	88.1%	87.4%	MH203213
	ISS91/Paz2014	86.1%	86.2%	86.2%	MH203212
**3e**	ISS83/Paz2014	81.6%	81.9%	80.8%	MH203208
	ISS51/Paz2014	81.2%	81.5%	80.5%	MH203190
	ISS20/Plov2013	80.8%	81.2%	80.1%	MH203172
	ISS60/Plov2014	80.6%	80.5%	80.5%	MH203195
	ISS87/Sof2014	80.6%	81.5%	80.8%	MH203211
**3f**	ISS98/Paz2014	84.9%	85.0%	83.5%	MH203218
	ISS104/Bur2014	84.9%	84.6%	82.4%	MH203223
	ISS79/Sof2014	84.5%	84.2%	82.0%	MH203207
	ISS32/Jam2013	82.4%	82.3%	80.1%	MH203178
	ISS85/Paz2014	82.5%	82.8%	80.9%	MH203210
**3i**	ISS62/Paz2014	87.5%	87.4%	87.1%	MH203197

## Data Availability

The data that support the findings of this study are openly available in the GenBank database at https://www.ncbi.nlm.nih.gov/nucleotide/ under accession numbers MZ519907-MZ519915 for RdRp region and MZ555941-MZ555944 for the partial ORF2 gene. Data were submitted on 09 July 2021, BankIt submission no 2480254.
